# Assessing the Appropriateness of Antimicrobial Prescribing in the Community Setting: A Scoping Review

**DOI:** 10.1093/ofid/ofad670

**Published:** 2024-03-22

**Authors:** Rose I Okonkwo, Gary Grant, Henry Ndukwe, Zabiuddin Ahad Mohammed, Sohil Khan

**Affiliations:** School of Pharmacy and Medical Sciences, Griffith University, Gold Coast, Australia; School of Pharmacy and Medical Sciences, Griffith University, Gold Coast, Australia; School of Pharmacy and Medical Sciences, Griffith University, Gold Coast, Australia; School of Pharmacy and Medical Sciences, Griffith University, Gold Coast, Australia; School of Pharmacy and Medical Sciences, Griffith University, Gold Coast, Australia; Manipal College of Pharmaceutical Sciences and Prasanna School of Public Health, Manipal Academy of Higher Education, Manipal, India

**Keywords:** antimicrobial stewardship, antimicrobial resistance, appropriateness, community setting, quality of antimicrobial prescribing

## Abstract

**Background:**

This scoping review examined the concept and scope of appropriateness of antimicrobial prescribing in the community setting and how it has been measured.

**Methods:**

Utilizing the Joanna Briggs Institute’s methodology, we appraised peer-reviewed articles and unpublished studies, focusing on the US, UK, Canada, and Australia, with no limit to date.

**Results:**

Four basic components of antimicrobial prescribing to be evaluated during assessment of antimicrobial appropriateness in the community setting were identified: diagnosis for infection or indication for antimicrobial therapy, choice of antimicrobial therapy, dosing, and duration of therapy. The benchmark for definition of appropriateness is crucial in assessing antimicrobial prescribing appropriateness. The use of recommended guidelines as a benchmark is the standard for appropriate antimicrobial therapy, and when necessary, susceptibility testing should be explored.

**Conclusions:**

Studies evaluating the appropriateness of antimicrobial prescribing should assess these components of antimicrobial prescribing, and this should be clearly stated in the aim and objectives of the study.

## INTRODUCTION

Antimicrobial resistance (AMR) is a significant threat to human health and a growing challenge globally [1]. It is a risk to patient safety as microorganisms develop mechanisms to resist the effectiveness of currently available antimicrobials that treat infections [2]. Unnecessary and inappropriate prescribing and the associated use of antimicrobials promote AMR, lengthen treatment times, increase costs, and cause hospitalization for conditions generally managed in the community [3]. Antimicrobials continue to be overused and misused as compared with the recommendations from evidence-based treatment guidelines. In the community setting, high levels of inappropriate antimicrobial prescribing and use are well documented [4, 5]. The majority of antimicrobial use occurs in the community setting, although the intensity of antimicrobial use is higher in hospitals [2]. AMR is reported more often in hospitals than the community setting, as there are variations in antimicrobial susceptibility testing practices and surveillance systems, among other factors. Numerous factors contribute to inappropriate antimicrobial prescribing in the community setting: comorbidity, diagnostic uncertainty, general practitioners’ perception of patients’ expectation for antibiotics, and patients’ demand for antibiotics [6]. Preventing the development and spread of antimicrobial-resistant organisms depends on our capacity to recognize and minimize inappropriate antimicrobial use.

Antimicrobial stewardship (AMS) is a proven approach that promotes optimization of antimicrobial use and a means to combat AMR [7]. Optimizing antimicrobial use through the implementation of AMS programs is one of the World Health Organization's strategic objectives of the Global Action Plan on AMR recommended at the World Health Assembly in May 2015 [1]. The need for more standardized measures for appropriate antimicrobial prescribing has increased as a result of these objectives. AMS programs should be centered on reducing unnecessary and inappropriate use of antimicrobials by prescribing according to evidence-based guidelines, which translates into appropriate use of medicines and which results in improved patient outcomes and patient safety, as well as reduced AMR and costs [8, 9]. The tracking and reporting of antimicrobial prescribing, use, outcomes, and AMR are considered essential components of AMS programs, and these are pivotal to identifying the opportunities for improvement and evaluating interventions [8, 10, 11]. An important factor for health systems aiming to enhance antimicrobial prescribing is how to measure and evaluate baseline performance and ongoing progress of providers performance in prescribing antimicrobials, which can be monitored using appropriateness of antimicrobial prescribing as a measure for antimicrobial prescribing performance [9, 12]. In an effort to contain AMR, AMS programs have been established to measure and improve the appropriateness of antimicrobial prescribing and use [7, 12]. However, there is a sparsity of studies primarily focused on assessing the appropriateness of antimicrobial prescribing in the community setting.

There is a scarcity of synthesized evidence on methodical approaches to define and assess appropriateness of antimicrobial prescribing as an empirical measure in the community setting. Therefore, a scoping review was considered to explore and summarize the available evidence in literature, clarify key concepts, examine how research is conducted on this topic, and identify knowledge gaps [13]. This scoping review seeks to examine the concept and scope of appropriateness of antimicrobial prescribing in the community setting and how it has been measured in this setting in terms of the basic components of antimicrobial prescribing evaluated for appropriateness and the benchmark for the definition of appropriateness. The knowledge gaps highlighted from this review would create opportunities for future research in promoting appropriateness of antimicrobial prescribing in the community setting.

## METHODS

This scoping review was conducted in accordance with the Joanna Briggs Institute’s methodology for scoping reviews [13].

### Eligibility Criteria

We defined community setting as a health facility, neither hospital based nor affiliated, located outside a hospital inpatient/outpatient setting or acute care setting (eg, emergency). Examples of such settings where antimicrobials are prescribed include care homes, long-term care facilities, old people's homes, nursing homes, assisted living facilities, aged care homes, general practices, and community-based (nonhospital) ambulatory care facilities. This definition specifically excludes data related to hospital settings where most AMS programs are typically established and effectively coordinated. Table 1 outlines the inclusion and exclusion of studies in the review.

**Table 1.** Inclusion and Exclusion Criteria

Criteria	Inclusion	Exclusion
Population	Primary studies or reports in the community setting that Aimed at assessing the appropriateness of antimicrobial prescribing or use Examined inappropriate or appropriate antimicrobial prescribing or use Investigated prescribing quality or prescribing^a^ patterns or rates of antimicrobial prescribing in compliance to treatment guidelines as a component of their objectives Studies from the United States, United Kingdom, Canada, and Australia were included. These countries^b^ were considered, as they have some similarities in their health care infrastructure and practice systems and a prescription is required to obtain antimicrobials. Studies included all ages.	Studies that included mixed settings (ie, other settings not captured in the inclusion criteria), such as emergency, inpatient, intensive care unit, hospice, hospital-affiliated outpatient Studies in dental, veterinary, or eye clinics or settings Studies that included mixed countries (ie, other countries not captured in the inclusion criteria)
Intervention	Studies included for this review focused on systemic anti-infectives, the standard collection of data for the Anatomical Therapeutic Chemical class J (anti-infectives for systemic use), which are usually presented internationally.	Studies that included immune sera, immunoglobulins, vaccines, and anti-infectives for veterinary use Studies that examined the impact of interventions on appropriateness or evaluated the appropriateness of antimicrobial prescribing following the implementation of interventions
Outcome	Components of antimicrobial prescribing assessed for appropriateness: (1) antimicrobial initiation (ie, diagnosis for infection or indication for antimicrobial therapy), (2) choice of antimicrobial therapy, (3) dosing, and (4) duration of therapy Benchmark for definition of appropriateness.	…
Comparison	…	Studies that conducted comparative evaluations of antimicrobial prescribing/use or estimates of appropriate antimicrobial prescribing/use Studies that compared the appropriateness of antibiotic prescribing for a condition or different conditions/groups

^a^Studies that examined prescribing patterns or rates of antimicrobial prescribing were included, as they could be clinical audits assessing compliance with treatment guidelines [14].

^b^The rationale was to gauge a broader understanding of practice gaps in these countries’ contexts. Future reviews could expand the scope for other contexts.

The Australian National Antimicrobial Prescribing Survey (NAPS) is a standardized auditing tool that is developed to support health facilities to evaluate the quantity and quality of antimicrobial prescribing [15]. Five NAPS audits are currently available: hospital, surgical, antifungal, quality improvement, and aged care. The Hospital NAPS has been utilized in countries such as Canada, New Zealand, the United Kingdom, Bhutan, Malaysia, Nepal, Pakistan, Papua New Guinea, Fiji, Timor-Leste, and Vietnam, with a planned pilot in Portugal [15]. We adapted the Hospital NAPS appropriateness definitions for the context of the community setting and define an antimicrobial prescription as *appropriate* when it follows recommended guidelines optimally or covers the likely causative organism and there is not a narrower or more appropriate choice available. Likewise, we define an antimicrobial prescription as *inappropriate* when it was an unreasonable or unlikely choice to treat the likely causative organism, or the documented indication does not require antimicrobial treatment, or there may be a severe life-threatening allergy mismatch or the potential risk of toxicity due to drug interaction [16]. For this review, we suggested that the basic components of antimicrobial prescribing assessed for appropriateness include antimicrobial initiation (ie, diagnosis for infection or indication for antimicrobial therapy), choice of antimicrobial therapy, dosing, and duration of therapy. The benchmark for definition of appropriateness of each of these components should be based on recommended guidelines and, where necessary, the antimicrobial choice determined by the susceptibility test. Therefore, the criteria for assessment of appropriateness of antimicrobial prescribing in the community setting should include the assessment of all the basic components of antimicrobial prescribing.

### Search Strategy

The search involved scanning electronic databases and gray literature sources for peer-reviewed articles and unpublished studies, respectively. An initial limited search of MEDLINE (Ovid) and CINAHL was undertaken. The related keywords from titles and abstracts of relevant articles and the index terms used to describe the articles were developed to a full search strategy to perform electronic searches in the following bibliographic databases: Cochrane Central Register of Controlled Trials, MEDLINE (Ovid), Embase (Ovid), CINAHL, and the Web of Science (Supplementary material). The search strategy, including all identified keywords and index terms, was adapted for each database and had no limit to age, date, and language. The reference lists of all included sources of evidence was screened for additional studies. A citation search in the Web of Science was used to identify other potentially relevant studies. Sources of gray literature consisted of clinical and practice guidelines; government reports; the Google search engine; and the websites of the Australian Commission on Safety and Quality in Health Care, Royal Australian College of General Practitioners, European Centre for Disease Prevention and Control, England's National Health Service, National Institute for Health and Care Excellence, Royal College of General Practitioners, British Society for Antimicrobial Chemotherapy, US Centers for Disease Control and Prevention, Government of Canada, and World Health Organization. Such works were included if they met the inclusion and exclusion criteria. An evaluation and critical appraisal tool—the AACODS checklist (authority, accuracy, coverage, objectivity, date, significance) [17]—was used for evaluating gray literature.

### Evidence Selection

Following the search, identified citations were collated and uploaded into EndNote version 20 (Clarivate Analytics) and imported into the Covidence website, where the system automatically removed duplicates. Within the Covidence website, articles were filtered by a member of the research team (R. I. O.), and any further duplicates were removed manually. Following a pilot test, titles and abstracts were screened by R. I. O. for assessment against the inclusion criteria for the review. Potentially relevant sources were selected and transferred into the full-text review section. The full texts of selected citations were independently assessed in detail against the inclusion criteria by R. I. O. and H. N. Reasons for exclusion were recorded and reported. Any disagreements between the reviewers during the selection process were resolved through discussions with a third member of the research team (S. K.) to make a final decision on including an article or not. The results of the search and the study inclusion process are reported in full and presented per the PRISMA-ScR flow diagram (Preferred Reporting Items for Systematic Reviews and Meta-analyses extension for scoping reviews) [18].

### Data Extraction and Presentation

We used a standardized data extraction tool that included details about author, year, title, reference, country, setting, study design, components of antimicrobial prescribing evaluated for appropriateness (diagnosis for infection or indication for antimicrobial therapy, choice of therapy, dosing, and duration), and benchmark for the definition of appropriateness in each study (Table 2).

**Table 2.** Studies That Evaluated the Appropriateness of Antimicrobial Prescribing and Use

				Components of Antimicrobial Prescribing Evaluated for Appropriateness					
					Choice of Therapy				
First Author (Year)	Country	Setting	Study Design	Diagnosis or Indication	Empiric	Definitive	Dosing	Duration	Benchmark for Definition of Appropriateness
D’Achille (1981) [19]	USA	Primary care	Retrospective cohort study	✓	✓	✓	✓	✓	Published literature and susceptibility test
Zimmer (1986) [20]	USA	Nursing homes, long-term care facilities, intermediate care facilities	Retrospective study	✓	✓				Authors (expert) opinion, guidelines, Food and Drug Administration–approved indications in manufacturer's package inserts^a^
Jones (1987) [21]	USA	Nursing homes	Retrospective study	✓	✓				Published studies
Montgomery (1995) [22]	Canada	Nursing homes/long-term care facilities	Retrospective study	✓	✓	✓			Published study, expert opinion, and susceptibility test
Arnold (1999) [23]	Canada	Primary care	Retrospective study	✓					Guidelines and expert opinion; susceptibility test
Nash (2002) [24]	USA	Primary care	Retrospective study	✓	✓	✓			Guidelines and susceptibility test
Takahashi (2002) [25]	USA	Nursing home residents and independent living in the community	Retrospective cohort study				✓		Guidelines
Arnold (2006) [26]	USA	Primary care pediatric setting	Retrospective study	✓					Guidelines
Vergidis (2011) [27]	USA	Nursing homes/long-term care facilities	Retrospective cohort study	✓	✓				Guidelines and published recommendations
Rotjanapan (2011) [28]	USA	Nursing homes	Retrospective cohort study	✓	✓		✓	✓	Guidelines and published recommendations
Stuart (2012) [29]	Australia	Residential aged care facilities	Point prevalence survey, retrospective review	✓					Published study (McGeer criteria)
Kistler (2017) [30]	USA	Community-based nursing homes	Retrospective cohort study	✓					Guidelines and published recommendations; susceptibility test
Eure (2017) [31]	USA	Nursing home	Point prevalence survey, retrospective review	✓					Published algorithms (revised McGeer criteria, Loeb minimum criteria, Crnich algorithm) and published recommendations (including susceptibility test)
Smith (2018) [32]	UK	Primary care	Retrospective study	✓					Guidelines and expert elicitation
Singer (2018) [33]	Canada	Community-based primary care practices	Retrospective cohort study	✓	✓			✓	Guidelines and author opinion
Smieszek (2018) [34]	UK	Primary care	Retrospective cohort study	✓					Guidelines and expert opinion
Shively (2018) [35]	USA	Primary care	Retrospective cohort study	✓	✓			✓	Guidelines and author (expert) opinion
Stuart (2020) [36]	UK	Primary care	Secondary analysis of 3 large primary care cohorts, retrospective review	✓					Guidelines and published studies
Howarth (2020) [37]	Australia	Remote Aboriginal communities	Retrospective cohort study	✓	✓				Guidelines
Truitt (2021) [38]	USA	Urban, suburban, and rural primary care practices	Retrospective study	✓	✓	✓			Guidelines, author opinion and susceptibility test
de Jong (2021) [39]	Australia	Aboriginal community controlled primary health care remote clinics	Retrospective observational study	✓	✓		✓	✓	Guidelines
Li (2021) [40]	UK	5 general practice surgeries (primary care setting)	Retrospective observational study	✓					Guidelines and author opinion
Mullakary (2021) [41]	USA	Primary care setting (health centers and care for the elderly)	Retrospective cohort study	✓	✓	✓	✓	✓	Guidelines and susceptibility test
Australian Commission on Safety and Quality in Health Care (2016) [42]	Australia	Aged care	Point prevalence survey, retrospective review	✓				✓	Guideline, McGeer criteria^a^, and microbiology test
Australian Commission on Safety and Quality in Health Care (2017) [43]	Australia	Aged care	Point prevalence survey, retrospective review	✓				✓	Guideline, McGeer criteria^a^, and microbiology test
Australian Commission on Safety and Quality in Health Care (2018) [44]	Australia	Aged care	Point prevalence survey, retrospective review	✓				✓	Guideline, McGeer criteria^a^, and microbiology test
Australian Commission on Safety and Quality in Health Care (2019) [45]	Australia	Aged care	Point prevalence survey, retrospective review	✓				✓	Guideline, McGeer criteria^a^, and microbiology test
Australian Commission on Safety and Quality in Health Care (2020) [46]	Australia	Aged care	Point prevalence survey, retrospective review	✓				✓	Guideline, McGeer criteria^a^, and microbiology test
Australian Commission on Safety and Quality in Health Care (2016) [47]	Australia	General practices	Retrospective review	✓	✓				Guideline
Australian Commission on Safety and Quality in Health Care (2017) [48]	Australia	General practices	Retrospective review	✓	✓				Guideline
Australian Commission on Safety and Quality in Health Care (2019) [49]	Australia	General practices	Retrospective review	✓	✓				Guideline
Australian Commission on Safety and Quality in Health Care (2021) [2]	Australia	General practices	Retrospective review	✓	✓				Guideline
Australian Commission on Safety and Quality in Health Care (2022) [50]	Australia	General practices	Retrospective review	✓	✓				Guideline

^a^Food and Drug Administration and McGeer benchmarks are based on published studies.

## RESULTS

### Literature Search

**Figure 1.**
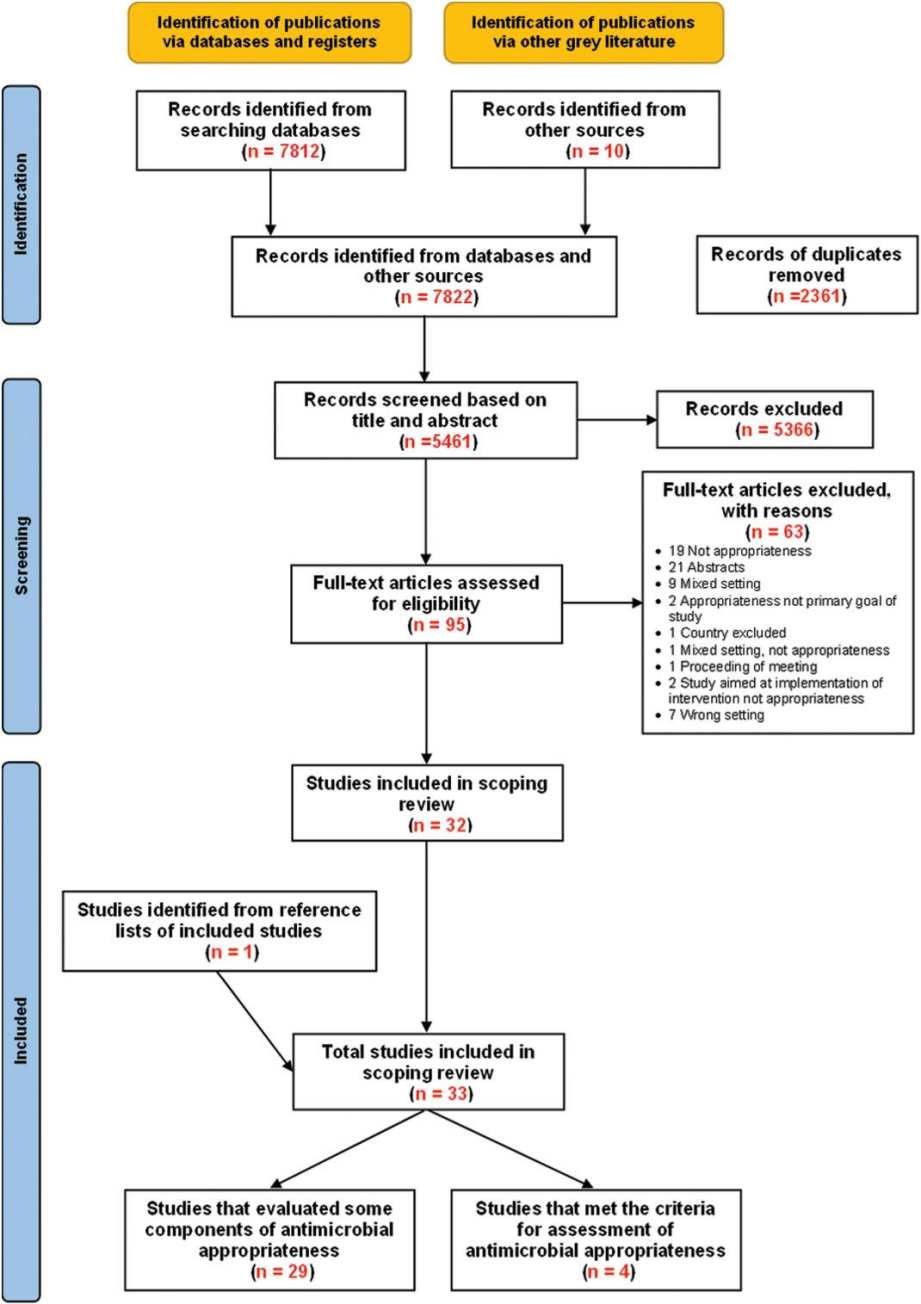
PRISMA-ScR flow diagram (Preferred Reporting Items for Systematic Reviews and Meta-analyses extension for scoping reviews).

In total, 7822 articles were identified during the initial search, of which 5461 were screened for title and abstracts after removal of 2361 duplicates. A total of 5366 studies were excluded by title and abstract screening. Full-text articles were retrieved for the remaining 95 studies. From these, 63 studies were excluded, and reasons for their exclusion are provided in Figure 1. The reference lists of the 32 studies were screened for additional studies, and 1 was identified and included in the review for a final total of 33 studies.

### Characteristics of the Articles

The studies were published from 1981 to 2022, with about two-thirds published in the last 5 years (Table 2). Of the 33 studies, 23 were from peer-reviewed journal articles and 10 from gray literature sources. Of the 23 studies from peer-reviewed journal articles, 13 were conducted in the United States [19–21, 24–28, 30, 31, 35, 38, 41], 3 in Australia [29, 37, 39], 4 in the United Kingdom [32, 34, 36, 40], and 3 in Canada [22, 23, 33]. All 10 studies from gray literature sources were from Australia [2, 42–50]. All 33 studies were retrospective—either cohort or point prevalence surveys. Nineteen studies were in primary care, community health care clinic, or general practice settings [2, 19, 23, 24, 26, 32–41, 47–50], while 14 were in nursing homes, long-term care facilities, or aged care settings [20–22, 25, 27–31, 42–46]. A description of studies is provided in Table 2.

### Components of Antimicrobial Prescribing Evaluated for Appropriateness

Of the 33 studies, 32 assessed appropriateness of diagnosis for infection or indication for antimicrobial therapy [2, 19–24, 26–50] (Table 3). Eighteen studies evaluated appropriateness of the choice of antimicrobial therapy [2, 19–22, 24, 27, 28, 33, 35, 37–39, 41, 47–50], and of these, 5 assessed empiric and definitive drug selection [19, 22, 24, 38, 41]. Assessment of antimicrobial dosing appropriateness was done in 5 of 33 studies [19, 25, 28, 39, 41], and evaluation of antimicrobial duration appropriateness was conducted in 11 of 33 studies [19, 28, 33, 35, 39, 41–46]. Of the 33 studies, 4 evaluated all the components of antimicrobial prescribing appropriateness; specifically, they met the criteria for assessment of antimicrobial prescribing appropriateness, which includes the diagnosis for infection or indication for antimicrobial therapy, choice of antimicrobial therapy, dosing, and duration of therapy [19, 28, 39, 41]. Of these 4 articles, 2 evaluated empiric and definitive therapy as a component of choice of antimicrobial therapy [19, 41], and 1 evaluated daily dose and dosage interval as a component of appropriateness of antimicrobial dosing [19].

**Table 3.** Studies That Evaluated the Appropriateness of Antimicrobial Prescribing and Use: Proportion of Studies That Assessed 1 or All Components of Antimicrobial Prescribing Appropriateness

References	Components of Antimicrobial Prescribing Evaluated for Appropriateness	No. (%)^a^
2, 19–24, 26–50	Antimicrobial initiation—specifically, diagnosis for infection or indication for antimicrobial therapy	32 (97.0)
2, 19–22, 24, 27, 28, 33, 35, 37–39, 41, 47–50	Choice of antimicrobial therapy	18 (54.5)
19, 25, 28, 39, 41	Antimicrobial dosing	5 (15.5)
19, 28, 33, 35, 39, 41–46	Duration of treatment	11 (33.3)
19, 28, 39, 41	Antimicrobial initiation, choice of antimicrobial therapy, antimicrobial dosing, and duration of treatment	4 (12.1)

^a^Out of 33 total studies.

### Benchmark for Definition of Appropriateness

Most of the studies assessing appropriateness of antimicrobial therapy in the community setting defined appropriateness based on recommended guidelines [2, 20, 23–28, 30, 32–50] (Table 4). Some defined appropriateness based on published studies or recommendations [19–22, 27–31, 36, 42–46], susceptibility tests [19, 22–24, 30, 31, 38, 41–46], and an expert or author's opinion [20, 22, 23, 32–35, 38, 40]. Of the 4 studies that met the criteria for assessment of antimicrobial prescribing appropriateness, 3 defined appropriateness based on recommended guidelines, including other parameters [28, 39, 41], and the remaining 1 study defined appropriateness based on published literature and other considerations [19]. Susceptibility tests were conducted in 2 of the 4 studies [19, 41].

**Table 4.** Studies That Evaluated the Appropriateness of Antimicrobial Prescribing and Use: Proportion of Studies That Assessed by Benchmark for Definition of Appropriateness

References	Benchmark for Definition of Appropriateness	No. (%)^a^
2, 20, 23–28, 30, 32–50	Recommended guidelines	28 (84.8)
19, 22–24, 30, 31, 38, 41–46	Susceptibility tests	13 (39.4)
19–22, 27–31, 36, 42–46	Published studies or recommendations	15 (45.5)
20, 22, 23, 32–35, 38, 40	Expert and/author opinion	9 (27.3)

^a^Out of 33 total studies.

## DISCUSSION

This scoping review appraised studies that assessed the appropriateness of antimicrobial prescribing and use in the community setting. According to the adapted definition, most studies did not meet the criteria for assessment, as the components of antimicrobial prescribing were not assessed by a holistic approach of assessing all 4 basic components. The majority of the studies evaluated the appropriateness of antimicrobial initiation, which is the first step that must be undertaken. Understanding the purpose for an antimicrobial being prescribed is key to assessing prescribing appropriateness. The benchmark for definition of appropriateness is crucial in assessing antimicrobial prescribing appropriateness. Most studies applied recommended guidelines as a benchmark, which is the standard for appropriate therapy and a key indicator for assessment. It reduces bias and provides a reliable method of measurement within and across health facilities; therefore, this allows for comparison of data and implementation of sustainable AMS strategies across health facilities. Using susceptibility tests as benchmark for definition of appropriateness was reported in a considerable number of studies. It is a definitive choice of antimicrobial therapy and an objective criterion with high clinical significance [51]. Utilizing published studies to define appropriateness is less subjective than definitions based on expert opinion because they are peer reviewed. Expert opinions, though clinically acceptable, are not standardized and not a reliable method across health facilities; thus, it is difficult to compare their data. It is important to note that in comparison with the hospital setting, the community setting is perceived to be more complex; oftentimes, infections and implicated microorganisms are not identified before general practitioners make the decision to prescribe or not prescribe, and sometimes patients are not followed up with further treatment plans to change or discontinue antibiotics. The benchmark for definition of appropriateness during the assessment of antimicrobial prescribing appropriateness should be obtained from applicable recommended guidelines and, when required, the antimicrobial choice defined by susceptibility testing.

The effectiveness of AMS programs can be evaluated by using quality indicators or measures. Recommended key measures of performance measurement and evaluation are structure, process, outcome, and balancing measures [9]. Assessing the quality of antimicrobial prescribing is a significant part of AMS programs and can be achieved by measuring and evaluating process measures that determine whether policies and practices are being followed correctly. Examples of process measures relating to the quality of antimicrobial prescribing that are associated with the prescription include antimicrobial appropriateness, rates of adherence to recommended guidelines, compliance to antimicrobial restriction conditions, and antimicrobial prescribing rates of varying diseases [9, 12]. Appropriateness of antimicrobial prescribing is a better measure than antimicrobial consumption, and it has been described as a gold standard metric for measuring effectiveness of AMS programs [52]. Process measures such as appropriateness can be effective methods that help to maintain prescribing performance at an appropriately high level when established as regular audits and feedback provided to prescribers and other stakeholders [9]. Evidence shows that appropriate antimicrobial prescribing and AMS activities have reported improved outcomes, such as improved patient outcomes and safety, as well as reduced AMR and costs [8, 53]. Nonetheless, health facilities may find it more feasible to evaluate process measures that can be reliably linked to improvement in outcomes and utilize them as proxies for outcome measures [9].

In Australia's residential aged care facilities, the 2 key indicators for assessing antimicrobial prescribing appropriateness monitored through the Aged Care NAPS are documentation of indication (best practice >95%) and documenting a review or stop date (best practice >95%) [46]. In addition, the revised McGeer infection criteria are used as a measure of appropriateness of antimicrobial prescribing initiation [46, 54]. Antimicrobials prescribed for residents with signs, symptoms, and investigations that meet the McGeer criteria are deemed “appropriate.” The best practice target of >95% for documentation of indication is based on the European Surveillance of Antimicrobial Consumption’s point prevalence survey, designed by the European Centre for Disease Prevention and Control [55]. There is no published best practice target for documenting a review or stop date; however, the Australian Antimicrobial Stewardship Clinical Care Standard requires that all prescriptions have the intended duration and review plan documented in the health record [56]. Hence, the same best practice target of >95% was used for this indicator. The General Practice NAPS has been piloted, and the key indicators for assessing the appropriateness of antimicrobial prescribing are documentation of antimicrobial prescribing components in the electronic medical record, compliance with evidence-based guidelines, and appropriateness. There is evidence to support the reliability of the General Practice NAPS [57]. The General Practice NAPS promotes AMS as a health care delivery component in primary care, general practices, and outpatient settings. In the hospital setting, the key indicators for assessing appropriateness of antimicrobial prescribing monitored through the Hospital NAPS are documentation of indication (best practice >95%), documentation of review or stop date (best practice >95%), surgical prophylaxis given for >24 hours (best practice <5% where surgical prophylaxis was selected as the indication), compliance with evidence-based guidelines (categorized as compliant, noncompliant, directed therapy, no guideline available, and not assessable), and appropriateness (which may be categorized as appropriate, inappropriate, and not assessable) [16]. The Hospital NAPS evaluates all the components of antimicrobial prescribing appropriateness.

Identified studies show that evidence is weak in the community setting. The community setting is not as controlled as the hospital setting, as there are peculiar practical difficulties in the community setting, such as documentation, patient loss to follow-up, logistics, and timeliness of susceptibility tests, among others. A retrospective cohort study conducted in the United States that determined the appropriateness of antibiotic initiation, selection, dosing, and duration of treatment among patients in nursing homes demonstrated that all quality indicators and components of antimicrobial prescribing appropriateness in the community setting were applicable in residential aged care facilities and that assessing all the components of antimicrobial prescribing appropriateness is achievable in this context [28]. Evidence shows its applicability in various contexts in the community setting [19, 28, 39, 41]. Therefore, studies evaluating the appropriateness of antimicrobial prescribing in the community setting should assess all basic components of antimicrobial prescribing: appropriateness of antimicrobial initiation, drug selection, dosing, and duration of treatment. It should also be clearly stated in the aim and objectives of the study. For example, if a study assesses diagnosis for infection or indication for antimicrobial therapy only, it should be stated and aimed at appropriateness of antimicrobial initiation. This should apply to the choice of therapy, dosing, and duration and stated as appropriateness of antimicrobial selection, dosing, and duration, respectively. This was demonstrated in 1 of the studies conducted in United States among nursing home residents and independent living in the community. This study evaluated antimicrobial dosing and stated it as one of its objectives [25]. However, another study that stated its aim to evaluate the appropriateness of antibiotic prescribing in primary care assessed only the appropriateness of antimicrobial initiation [40]. In all studies, outcomes of appropriateness measured were expressed as percentages of appropriate or inappropriate prescriptions; these are process measures and can be used as substitutes for outcome measures, which may give insight into economic, clinical, and humanistic health outcomes. To the best of our knowledge, Australia is currently the only country in the world that evaluates and reports appropriateness of antimicrobial prescribing at a granular level nationally. Internationally distinctive, the Australian NAPS has showcased the feasibility and acceptability of measuring and assessing antimicrobial prescribing appropriateness in health systems for all antimicrobials being used, instead of a small number of selected drugs, and is currently utilized in the hospital and aged care settings [58]. According to the Centers for Disease Control and Prevention’s Implementation Resources for Outpatient Antibiotic Stewardship, health facilities have the option to utilize various data sources to assess antimicrobial prescribing appropriateness; nevertheless, some of the approaches used to evaluate outpatient antimicrobial prescribing lack the ability to assess appropriateness due to the absence of essential clinical information, which typically require electronic health records or linkage to insurance claims data [12]. It is worth emphasizing that the process measures or antimicrobial prescribing metrics recommended in the Centers for Disease Control and Prevention’s resource were specific to particular disease conditions, making it easier to track process measures and outcomes [12].

There are some potential limitations to this review. One reviewer (R. I. O.) conducted the screening by title and abstracts and the data extraction, which may have introduced selection bias. Also, scoping reviews do not assess the quality of the evidence, and no implications for policy are recommended [13]. This scoping review may have limited applicability for community settings in countries where antimicrobials are obtained without a prescription. We focused on studies from the United States, United Kingdom, Canada, and Australia; therefore, findings are limited to understanding of practice gaps in these countries’ contexts. Review of audit tools and methods for evaluation of each component of antimicrobial prescribing appropriateness was beyond the scope of this research, and future studies should be considered. Furthermore, patient outcomes were not evaluated in this study.

This review demonstrated that few studies met the set criteria for evaluation of antimicrobial prescribing appropriateness, possibly because the evidence for appropriateness in the community setting is still emerging; thus, further research is required in this area. Retrospective review of medical records is the most common approach used to evaluate the appropriateness of antimicrobial prescribing and use in the community setting. Some of the assessment method and tools used in these studies have been used on a large scale, and findings have shown valuable information required for evidence-based AMS strategies to improve antimicrobial prescribing. Standardized tools and use of advance electronic platforms, as well as exploring potentials of artificial intelligence in this area, could help secure large data on antimicrobial prescribing appropriateness through routine assessment on a national level. Investigating the role of artificial intelligence in detecting inappropriate prescribing through algorithms that automatically evaluate the basic components of antimicrobial prescribing appropriateness may be required. Research should consider methods that are sustainable and technologically informed assessments. Furthermore, studies aimed at institutionalization of antimicrobial prescribing appropriateness in the community setting should be encouraged.

## CONCLUSION

This review has attempted to define antimicrobial appropriateness on the basis of recommended guidelines and set out the basic components of antimicrobial prescribing to be evaluated for antimicrobial appropriateness in the community setting. Studies sought to assess some or all of the identified components of antimicrobial prescribing appropriateness in the community setting; however, no recognized reference standard exists. There is a lack of a standard protocol that clearly defines assessment of antimicrobial prescribing appropriateness in the community setting. Also, there is a sparsity of evidence within AMS regarding the antimicrobial prescribing appropriateness definition, particularly in the community setting. Within these limitations, we propose the following as basic components of antimicrobial prescribing to be evaluated during assessment of antimicrobial appropriateness in the community setting: diagnosis for infection or indication for antimicrobial therapy, choice of antimicrobial therapy, dosing, and duration of therapy. Most studies assessing the appropriateness of antimicrobial therapy in the community setting have used evidence-based guidelines as a benchmark for definition of appropriateness, and findings have shown important information needed to enhance antimicrobial prescribing and use. Community-level AMS implementation is more challenging as compared with that in the hospital setting, and this review has highlighted the need for the development and standardization of process measures and resources to implement assessment of antimicrobial prescribing appropriateness in the community setting. Surveillance on appropriateness of antimicrobial prescribing and use is essential to inform AMR prevention and containment strategies. It should be encouraged that health facilities where antimicrobials are prescribed should routinely conduct assessment of appropriateness of antimicrobial prescribing.

## Supplementary Material

ofad670_Supplementary_Data

## Supplementary Data

Supplementary materials are available at *Open Forum Infectious Diseases* online. Consisting of data provided by the authors to benefit the reader, the posted materials are not copyedited and are the sole responsibility of the authors, so questions or comments should be addressed to the corresponding author.

## Notes

***Author contributions.*** R. I. O.: conception and design, analysis, and interpretation of the data; drafting of the manuscript and critical revision for important intellectual content; and final approval of the version to be published. G. G., H. N.: analysis and interpretation of the data, critical revision for important intellectual content, and final approval of the version to be published. Z. A. M.: interpretation of the data, critical revision for important intellectual content, and final approval of the version to be published. S. K.: design, analysis, and interpretation of the data; critical revision for important intellectual content; and final approval of the version to be published.

***Patient consent statement.*** This study does not include factors necessitating patient consent.

***Financial support.*** This work was supported by the Griffith University Postgraduate Research Scholarship and Griffith University International Postgraduate Research Scholarship.
