# Supplementary material for: Assessing the Appropriateness of Antimicrobial Prescribing in the Community Setting: A Scoping Review
Source: Open Forum Infect Dis. 2024 Mar 22;11(3):ofad670. doi: 10.1093/ofid/ofad670 (PMC10959551; doi:10.1093/ofid/ofad670)
Supplement: ofad670_Supplementary_Data [file ofad670_supplementary_data.docx]

**SEARCH STRATEGY**

**DATABASE: MEDLINE (OVID)**

Ovid MEDLINE(R) <1946 to March 15, 2023>

| **#** | **Query** | **Results from 15 Mar 2023** |
| --- | --- | --- |
| 1 | ("antimicrobial* use" or "anti-microbial* use" or "antibiotic* use" or "anti-biotic* use" or "antibacterial* use" or "anti-bacterial* use" or "antifungal* use" or "anti-fungal* use" or "antiviral* use" or "anti-viral* use" or "anti-infective* use" or "antiinfective* use").ti,ab. | 15,005 |
| 2 | ((antimicrobial* or anti-microbial* or antibiotic* or anti-biotic* or antibacterial* or anti-bacterial* or antifungal* or anti-fungal* or antiviral* or anti-viral* or anti-infective* or antiinfective*) adj2 prescri*).ti,ab. | 12,238 |
| 3 | ((antimicrobial* or anti-microbial* or antibiotic* or anti-biotic* or antibacterial* or anti-bacterial* or antifungal* or anti-fungal* or antiviral* or anti-viral* or anti-infective* or antiinfective*) adj2 (quality or appropriate* or inappropriate* or optimis* or optimiz* or optimal or guideline* or pattern*)).ti,ab. | 23,026 |
| 4 | exp Residence Characteristics/ | 77,271 |
| 5 | exp residential facilities/ or exp General Practice/ or exp family practice/ or exp community health centers/ or exp community health services/ or exp home care services/ or exp health services for the aged/ or exp physicians' offices/ or exp ambulatory care/ or exp outpatient clinics/ or exp community health nursing/ or exp home nursing/ or exp pharmacies/ or exp community pharmacy services/ | 574,225 |
| 6 | ("community practice" or "community pharmacy" or "community setting*" or "community care" or "community health centre*" or "non-hospital setting*" or "care home*" or "old age home*" or "elderly care home*" or "aged care home*" or "nurs* home*" or "primary care" or "primary health care" or "primary healthcare" or "out-patient clinic").ti,ab. | 197,610 |
| 7 | 1 or 2 | 24,407 |
| 8 | 3 and 7 | 4,540 |
| 9 | 4 or 5 or 6 | 771,988 |
| 10 | 8 and 9 | 905 |

**DATABASE: EMBASE (OVID)**

18 Mar 2023

| **#** | **Query** | **Results from 18 Mar 2023** |
| --- | --- | --- |
| #9 | #7 AND #8 | 4,619 |
| #8 | #4 OR #5 | 2,528,924 |
| #7 | #3 AND #6 | 8,966 |
| #6 | #1 OR #2 |  |
| #5 | 'community practice':ti,ab OR 'community pharmacy':ti,ab OR 'community setting*':ti,ab OR 'community care':ti,ab OR 'community health centre*':ti,ab OR 'non-hospital setting*':ti,ab OR 'care home*':ti,ab OR 'old age home*':ti,ab OR 'elderly care home*':ti,ab OR 'aged care home*':ti,ab OR 'nurs* home*':ti,ab OR 'primary care':ti,ab OR 'primary health care':ti,ab OR 'primary healthcare':ti,ab OR 'out-patient clinic':ti,ab | 44,332 |
| #4 | 'pharmacy (shop)'/exp OR 'community health nursing'/exp OR 'outpatient department'/exp OR 'ambulatory care'/exp OR 'health care facility'/exp OR 'elderly care'/exp OR 'home care'/exp OR 'community care'/exp OR 'health center'/exp OR 'general practice'/exp OR 'residential home'/exp OR 'residence characteristics'/exp | 302,596 |
| #3 | ((antimicrobial* OR 'anti-microbial*' OR antibiotic* OR 'anti biotic*' OR antibacterial* OR 'anti-bacterial*' OR antifungal* OR 'anti-fungal*' OR antiviral* OR 'anti-viral*' OR 'anti-infective*' OR antiinfective*) NEAR/2 (quality or appropriate* OR inappropriate* OR optimis* OR optimiz* OR optimal OR guideline* OR pattern*)):ti,ab | 2,348,069 |
| #2 | ((antimicrobial* OR 'anti-microbial*' OR antibiotic* OR 'anti biotic*' OR antibacterial* OR 'anti-bacterial*' OR antifungal* OR 'anti-fungal*' OR antiviral* OR 'anti-viral*' OR 'anti-infective*' OR antiinfective*) NEAR/2 prescri*):ti,ab | 40,918 |
| #1 | 'antimicrobial* use':ti,ab OR 'anti-microbial* use':ti,ab OR 'antibiotic* use':ti,ab OR 'anti-biotic* use':ti,ab OR 'antibacterial* use':ti,ab OR 'anti-bacterial* use':ti,ab OR 'antifungal* use':ti,ab OR 'anti-fungal* use':ti,ab OR 'antiviral* use':ti,ab OR 'anti-viral* use':ti,ab OR 'anti-infective* use':ti,ab OR 'antiinfective* use':ti,ab | 22,667 |

**DATABASE: Web of Science**

18032023

| **#** | **Query** | **Results from 18 Mar 2023** |
| --- | --- | --- |
| 9 | #7 AND #8 | 1,511 |
| 8 | #4 OR #5 | 327,500 |
| 7 | #3 AND #6 | 7,129 |
| 6 | #1 OR #2 | 32,022 |
| 5 | TS=("community practice" OR "community pharmacy" OR "community setting*" OR "community care" OR "community health centre*" OR "non-hospital setting*" OR "care home*" OR "old age home*" OR "elderly care home*" OR "aged care home*" OR "nurs* home*" OR "primary care" OR "primary health care" OR "primary healthcare" OR "out-patient clinic") | 261,485 |
| 4 | TS=("residence characteristics" OR "residential facilities" OR "general practice" OR "family practice" OR "community health centers" OR "community health services" OR "home care services" OR "health services for the aged" OR "physicians' offices" OR "ambulatory care" OR "outpatient clinics" OR "community health nursing" OR "home nursing" OR "pharmacies" OR "community pharmacy services") | 92,105 |
| 3 | TS=(((antimicrobial* or anti-microbial* or antibiotic* or anti-biotic* or antibacterial* or anti-bacterial* or antifungal* or anti-fungal* or antiviral* or anti-viral* or anti-infective* or antiinfective*) Near/2 (quality or appropriate* or inappropriate* or optimis* or optimiz* or optimal or guideline* or pattern*))) | 31,560 |
| 2 | TS=(((antimicrobial* or anti-microbial* or antibiotic* or anti-biotic* or antibacterial* or anti-bacterial* or antifungal* or anti-fungal* or antiviral* or anti-viral* or anti-infective* or antiinfective*) Near/2 prescri*)) | 15,587 |
| 1 | TS=(("antimicrobial* use" or "anti-microbial* use" or "antibiotic* use" or "anti-biotic* use" or "antibacterial* use" or "anti-bacterial* use" or "antifungal* use" or "anti-fungal* use" or "antiviral* use" or "anti-viral* use" or "anti-infective* use" or "antiinfective* use")) | 20,405 |

**DATABASE: Cochrane Central Register of Controlled Trials (CENTRAL),**

Date Run: 18/03/2023 12:37:52

| **#** | **Query** | **Results from 18 Mar 2023** |
| --- | --- | --- |
| #1 | (("antimicrobial* use" or "anti-microbial* use" or "antibiotic* use" or "anti-biotic* use" or "antibacterial* use" or "anti-bacterial* use" or "antifungal* use" or "anti-fungal* use" or "antiviral* use" or "anti-viral* use" or "anti-infective* use" or "antiinfective* use")):ti,ab,kw | 2,121 |
| #2 | (((antimicrobial* or anti-microbial* or antibiotic* or anti-biotic* or antibacterial* or anti-bacterial* or antifungal* or anti-fungal* or antiviral* or anti-viral* or anti-infective* or antiinfective*) Near/2 prescri*)):ti,ab,kw | 2,013 |
| #3 | (((antimicrobial* or anti-microbial* or antibiotic* or anti-biotic* or antibacterial* or anti-bacterial* or antifungal* or anti-fungal* or antiviral* or anti-viral* or anti-infective* or antiinfective*) Near/2 (quality or appropriate* or inappropriate* or optimis* or optimiz* or optimal or guideline* or pattern*))):ti,ab,kw | 1,383 |
| #4 | MeSH descriptor: [Residence Characteristics] explode all trees | 2,322 |
| #5 | MeSH descriptor: [Residential Facilities] explode all trees | 2,293 |
| #6 | MeSH descriptor: [General Practice] explode all trees | 2,878 |
| #7 | MeSH descriptor: [Family Practice] explode all trees | 2,242 |
| #8 | MeSH descriptor: [Community Health Centers] explode all trees | 670 |
| #9 | MeSH descriptor: [Community Health Services] explode all trees | 17,358 |
| #10 | MeSH descriptor: [Home Care Services] explode all trees | 2,826 |
| #11 | MeSH descriptor: [Health Services for the Aged] explode all trees | 537 |
| #12 | MeSH descriptor: [Physicians' Offices] explode all trees | 27 |
| #13 | MeSH descriptor: [Ambulatory Care] explode all trees | 4,047 |
| #14 | MeSH descriptor: [Ambulatory Care Facilities] explode all trees | 2,211 |
| #15 | MeSH descriptor: [Community Health Nursing] explode all trees | 388 |
| #16 | MeSH descriptor: [Home Nursing] explode all trees | 323 |
| #17 | MeSH descriptor: [Pharmacies] explode all trees | 182 |
| #18 | MeSH descriptor: [Community Pharmacy Services] explode all trees | 332 |
| #19 | (("community practice" or "community pharmacy" or "community setting*" or "community care" or "community health centre*" or "non-hospital setting*" or "care home*" or "old age home*" or "elderly care home*" or "aged care home*" or "nurs* home*" or "primary care" or "primary health care" or "primary healthcare" or "out-patient clinic")):ti,ab,kw | 28,730 |
| #20 | #1 OR #2 | 3,662 |
| #21 | #3 AND #20 | 514 |
| #22 | {OR #4-#19} | 54,229 |
| #23 | #21 AND #22 | 157 |

**DATABASE: CINAHL**

Saturday, March 18, 2023, 11:00:07 AM

| **#** | **Query** | **Limiters/Expanders** | **Last Run Via** | **Results** |
| --- | --- | --- | --- | --- |
| S9 | S7 AND S8 | Expanders - Apply equivalent subjects  Search modes -Boolean/Phrase | Interface - EBSCOhost Research Databases Search Screen - Advanced Search Database - CINAHL Complete | 620 |
| S8 | S4 OR S5 | Expanders - Apply equivalent subjects  Search modes -Boolean/Phrase | Interface - EBSCOhost Research Databases Search Screen - Advanced Search Database - CINAHL Complete | 810,125 |
| S7 | S3 AND S6 | Expanders - Apply equivalent subjects  Search modes -Boolean/Phrase | Interface - EBSCOhost Research Databases Search Screen - Advanced Search Database - CINAHL Complete | 1,996 |
| S6 | S1 OR S2 | Expanders - Apply equivalent subjects  Search modes -Boolean/Phrase | Interface - EBSCOhost Research Databases Search Screen - Advanced Search Database - CINAHL Complete | 10,246 |
| S5 | TI (("community practice" or community pharmacy" or community setting*" or" community care" or "community health centre*" or "non-hospital setting*" or "care home*"or "old age home*" or "elderly care home*" or "aged care home*" or "nurs* home*"or "primary care" or" primary healthcare" or "primary health care" or "out-patient clinic")) OR AB (("community practice" or "community pharmacy" or "community setting*"or "community care" or "community health centre*" or "non-hospital setting*" or "care home*"or "old age home*" or "elderly care home*" or "aged care home*" or "nurs* home*"or "primary care" or "primary healthcare" or "primary health care" or "out-patient clinic")) | Expanders - Apply equivalent subjects  Search modes -Boolean/Phrase | Interface - EBSCOhost Research Databases Search Screen - Advanced Search Database - CINAHL Complete | 144,074 |
| S4 | (MH "Residence Characteristics+") OR(MH "Residential Facilities+") OR (MH "Family Practice") OR(MH "Community Health Centers+") OR (MH "Community Health Services+") OR (MH "Home Health Care+")OR (MH "Health Services for Older Persons") OR(MH "Practitioner's Office") OR (MH "Ambulatory Care") OR(MH "Ambulatory Care Facilities+") OR (MH "Outpatient Service") OR(MH "Community Health Nursing+") OR (MH "Home Nursing") OR (MH "Pharmacy, Retail") OR (MH "Pharmacy Service+") | Expanders - Apply equivalent subjects  Search modes -Boolean/Phrase | Interface - EBSCOhost Research Databases Search Screen - Advanced Search Database - CINAHL Complete | 721,981 |
| S3 | TI (((antimicrobial*or anti-microbial* or antibiotic* or anti-biotic*or antibacterial* or anti-bacterial* or antifungal*or anti-fungal* or antiviral*or anti-viral* or anti-infective* or antiinfective*) N2 (quality or appropriate* or inappropriate* or optimis*or optimiz* or optimal or guideline* or pattern*))) OR AB (((antimicrobial*or anti-microbial* or antibiotic* or anti-biotic*or antibacterial* or anti-bacterial* or antifungal*or anti-fungal* or antiviral*or anti-viral* or anti-infective* or antiinfective*) N2 (quality or appropriate* or inappropriate* or optimis*or optimiz* or optimal or guideline* or pattern*))) | Expanders - Apply equivalent subjects  Search modes -Boolean/Phrase | Interface - EBSCOhost Research Databases Search Screen - Advanced Search Database - CINAHL Complete | 5,177 |
| S2 | TI (((antimicrobial* or anti-microbial* or antibiotic* or anti-biotic*or antibacterial* or anti-bacterial* or antifungal*or anti-fungal* or antiviral*or anti-viral* or anti-infective* or antiinfective*) N2 prescri*)) OR AB (((antimicrobial* or anti-microbial* or antibiotic* or anti-biotic*or antibacterial* or anti-bacterial* or antifungal*or anti-fungal* or antiviral*or anti-viral* or anti-infective* or antiinfective*) N2 prescri*)) | Expanders - Apply equivalent subjects  Search modes -Boolean/Phrase | Interface - EBSCOhost Research Databases Search Screen - Advanced Search Database - CINAHL Complete | 5,735 |
| S1 | TI (("antimicrobial* use" or "anti-microbial* use" or "antibiotic* use" or "anti-biotic* use" or "antibacterial* use" or "anti-bacterial* use" or "antifungal* use" or "anti-fungal* use" or "antiviral*use" or "anti-viral* use" or "anti-infective* use" or "antiinfective* use")) OR AB (("antimicrobial* use "or "anti-microbial* use" or "antibiotic* use" or "anti-biotic* use" or "antibacterial* use" or "anti-bacterial* use" or "antifungal* use" or "anti-fungal* use" or "antiviral*use" or "anti-viral* use" or "anti-infective* use" or "antiinfective* use")) | Expanders - Apply equivalent subjects  Search modes -Boolean/Phrase | Interface - EBSCOhost Research Databases Search Screen - Advanced Search Database - CINAHL Complete | 5,841 |
